# AMPKα2 Overexpression Reduces Cardiomyocyte Ischemia-Reperfusion Injury Through Normalization of Mitochondrial Dynamics

**DOI:** 10.3389/fcell.2020.00833

**Published:** 2020-08-27

**Authors:** Yuanyan Deng, Sainan Chen, Mingming Zhang, Chen Li, Jing He, Ying Tan

**Affiliations:** ^1^Department of Cardiology, Integrated Hospital of Traditional Chinese Medicine, Southern Medical University, Guangzhou, China; ^2^Department of Emergency Medicine, Nanfang Hospital, Southern Medical University, Guangzhou, China; ^3^Department of Burns, Nanfang Hospital, Southern Medical University, Guangzhou, China; ^4^Department of Cardiology, Foshan Hospital Affiliated with Southern Medical University (The Second People’s Hospital of Foshan), Foshan, China; ^5^Department of Critical Care Medicine, Nanfang Hospital, Southern Medical University, Guangzhou, China

**Keywords:** AMPKα2, cardiomyocytes, ischemia-reperfusion injury, mitochondrial dynamics, Sirt3/PGC1α signaling pathway

## Abstract

Cardiac ischemia-reperfusion (I/R) injury is associated with mitochondrial dysfunction. Recent studies have reported that mitochondrial function is determined by mitochondrial dynamics. Here, we hypothesized that AMPKα2 functions as an upstream mediator that sustains mitochondrial dynamics in cardiac I/R injury and cardiomyocyte hypoxia-reoxygenation (H/R) *in vitro*. To test this, we analyzed cardiomyocyte viability and survival along with mitochondrial dynamics and function using western blots, qPCR, immunofluorescence, and ELISA. Our results indicated that both AMPKα2 transcription and translation were reduced by H/R injury in cardiomyocytes. Decreased AMPKα2 levels were associated with cardiomyocyte dysfunction and apoptosis. Adenovirus-mediated AMPKα2 overexpression dramatically inhibited H/R-mediated cardiomyocyte damage, possibly by increasing mitochondrial membrane potential, inhibiting cardiomyocyte oxidative stress, attenuating intracellular calcium overload, and inhibiting mitochondrial apoptosis. At the molecular level, AMPKα2 overexpression alleviated abnormal mitochondrial division and improved mitochondrial fusion through activation of the Sirt3/PGC1α pathway. This suggests AMPKα2 contributes to maintaining normal mitochondrial dynamics. Indeed, induction of mitochondrial dynamics disorder abolished the cardioprotective effects afforded by AMPKα2 overexpression. Thus, cardiac I/R-related mitochondrial dynamics disorder can be reversed by AMPKα2 overexpression in a manner dependent on the activation of Sirt3/PGC1α signaling.

## Introduction

Myocardial infarction (MI) ranks the first place in the leading causes of death worldwide. Myocardial ischemia-reperfusion (I/R) injury refers to metabolic dysfunction caused by reperfusion of ischemic myocardial blood flow and aggravation of cardiomyocyte structural damage (Basalay et al., 2018), resulting in cell death and enlargement of infarction. This complication occurs during coronary artery bypass grafting reperfusion therapy, coronary artery thrombolysis, and percutaneous coronary intervention, which accelerates the incidence and mortality of cardio-cerebrovascular diseases (Muessig et al., 2020). Therefore, the reduction of myocardial ischemia-reperfusion injury remains an urgent and active research problem.

Cardiomyocytes contain abundant mitochondria, required for myocardial contraction and relaxation (Jiang et al., 2019). Mitochondrial dysfunction may underlie the cardiomyocyte damage induced by I/R injury (Sedighi et al., 2019; Wallert et al., 2019). For example, mitochondrial damage promotes cardiomyocyte oxidative stress through induction of reactive oxygen species (ROS) overloading (Kohlhauer et al., 2019). In addition, dysregulated mitochondria fail to produce sufficient ATP to sustain cardiomyocyte metabolism, resulting in decreased myocardial blood-pumping capacity (Maneechote et al., 2019). Meanwhile, mitochondria are the second largest calcium pool in cardiomyocytes and their dysfunction is associated with an increased resting calcium concentration, which correlates with myocardial stiffness and restricted diastolic function (Zhu et al., 2018a). Although injured mitochondria are timely removed through autophagy (Zhou et al., 2018e), namely mitophagy, irreparable mitochondria can trigger apoptosis and induce cardiomyocyte death. Increased levels Bax and decreased levels Bcl-2 are hallmarks of mitochondrial apoptosis activation and have been noted in the reperfused heart tissues (Yang et al., 2019b). Inhibition of mitochondria-related oxidative stress, mitochondria-induced intracellular calcium overload, and mitochondria-triggered cardiomyocyte apoptosis partially alleviates myocardial damage after cardiac I/R injury (Chen et al., 2019b; Wang et al., 2020b; Zhou and Toan, 2020).

According to the structure-function paradigm in biology, structure determines the function of systems from proteins to cells and organisms. Correspondingly, the functions of mitochondria are highly regulated by mitochondrial morphology (Aghaei et al., 2019). Indeed, mitochondria are highly dynamic organelles undergoing regular cycles of division and fusion, termed as “mitochondrial dynamics,” in order to maintain functional shapes, distribution, DNA heredity, protein communication, and nutrient exchange (Khan et al., 2020; Li et al., 2020b). Disturbed mitochondrial dynamics in cardiac I/R injury are characterized by decreased mitochondrial fusion and increased mitochondrial cleavage (Zhou et al., 2017c; Jin et al., 2018; Jang and Javadov, 2020), which might be an early predictor of mitochondrial dysfunction and cardiomyocyte death. At the molecular level, abnormalities in mitochondrial dynamics cause mitochondrial fragmentation, reduce mitochondrial membrane potential, augment mitochondrial ROS production, and trigger mitochondrial apoptosis (Zhou et al., 2018b, c; Yu et al., 2019; Wang et al., 2020a). Unfortunately, the upstream regulators of mitochondrial dynamics remain unknown in the context of cardiac I/R injury.

AMP-activated protein kinase (AMPK) is an enzyme that regulates mitochondrial energy metabolism (Hou et al., 2019). Heterotrimeric AMPK contains a catalytic α-subunit and regulatory β- and γ-subunits. Of note, AMPK has two α-type isozymes. The α1 subunit seems to be widely expressed, whereas the α2 subunit is highly expressed in the live and skeletal and cardiac muscles (Rinschen et al., 2019). Mitochondrial metabolism and function are regulated by AMPKα2 in the cardiovascular system. For example, mitophagy is activated by AMPKα2 in a manner dependent on the PINK1/Parkin pathway (Shires and Gustafsson, 2018). Heart failure is attenuated by AMPKα2 through inhibition of mitochondria-mediated cardiac remodeling (Wang et al., 2018). Mitochondria-related glucose metabolism (Park et al., 2017) and fatty acid β-oxidation (Stride et al., 2012) are positively handled by AMPKα2. Notably, a recent study reported that mitochondrial fusion could be enhanced by AMPKα2 in the setting of cardiac I/R injury (Kaljusto et al., 2008), suggesting a possible role played by AMPKα2 in regulating mitochondrial dynamics disorder. Here, we explored the molecular relationships between AMPKα2 and mitochondrial dynamics in cardiac I/R injury.

## Materials and Methods

### Cardiomyocyte Isolation and Hypoxia/Reoxygenation Injury Model

All animals were housed and treated in accordance with guidelines from the NIH and Institutional Animal Care And Use Committees (IACUC). Cardiomyocytes were isolated from mouse hearts by the Langendorff-based method, as previously described (Zhou et al., 2017d), with minor modifications. Male mice of 6–8 weeks of age (25–30 g) were used. In brief, after a quick removal of the heart from the chest, the aorta was retrogradely perfused at 37°C for 3 min with calcium-free Tyrode buffer (137 mmol/L NaCl, 5.4 mmol/L KCl, 1 mmol/L MgCl_2_, 10 mmol/L glucose, 10 mmol/L HEPES [pH 7.4], 10 mmol/L 2, 3-butanedione monoxime, and 5 mmol/L taurine) gassed with 100% O_2_. The enzymatic digestion was initiated by the addition of collagenase type B (300 U/mL; Worthington) and hyaluronidase (0.1 mg/mL; Worthington) to the perfusion solution. When the heart became swollen after 20 min of digestion, the left ventricle was quickly removed, cut into several chunks, and gently pipetted for 2 min in calcium-free Tyrode buffer with 5% BSA. The supernatant containing the dispersed myocytes was filtered through a cell strainer and gently centrifuged at 50 g for 1 min. Most myocytes settled to a pellet, while crude non-myocyte fraction remained in suspension. The non-myocyte fraction was further sorted and analyzed by FACS analysis. This procedure usually yielded ≥80% viable rod-shaped ventricular myocytes with clear sarcomere striations. Myocytes with obvious sarcolemmal blebs or spontaneous contractions were not used. H/R injury was established through 1-h hypoxia and 2-h reoxygenation, as previously described (Zhou et al., 2017a). To overexpress AMPKα2, adenovirus AMPKα2 were transfected into cardiomyocytes. To inhibit the activity of Sirt3, cardiomyocytes were incubated with 3-TYP (3 mM) before AMPKα2 transfection.

### Mitochondrial Morphology and Function

Cells were collected and fixed with glutaraldehyde-formaldehyde (2% formaldehyde and 2.5% glutaraldehyde in 0.1M sodium cacodylate buffer, pH 7.4) overnight. Sections were imaged using a JEOL 1200EX electron microscope (Harvard Medical School EM core facility). Mitochondrial size and density were measured using ImageJ. Oxygen consumption rate (OCR) was measured using the Cell Mito Stress Kit (Seahorses Biosciences, 103015) on a XF96e extracellular flux analyzer (Seahorses Biosciences). Data were normalized to cell number, measured by DAPI staining of culture plates (Honda et al., 2019). OCR was expressed as pmol/min/2,000 cells. Based on OCR changes after the addition of oligomycin (1 μm), FCCP (1 μm), or antimycin/rotenone (0.5 μm) in sequence, mitochondrial function metrics were calculated as described in the Cell Mito Stress Kit manual. ECAR was expressed as mpH/min/2,000 cells (Higgs et al., 2019).

To measure mitochondrial membrane potential (MMP), cells were incubated with 10 μm JC-1 (Life Technologies, T3168). Then, the cells were imaged on an Olympus FV1000 inverted laser scanning confocal microscope. Quantitative analysis was performed by flow cytometry using a Propel Laboratories Avalon cytometer with a 100 μm nozzle and standard GFP/RFP filter sets (Darido et al., 2018). The data were analyzed using FlowJo software. As a positive control for mitochondrial depolarization, cells were treated with 1 μm FCCP. The ADP/ATP ratio was measured with isolated cells after 3 and 7 days of culture, using the ADP/ATP Ratio Assay Kit (Bioluminescent).

### Production of Adenovirus-AMPKα2

Adenovirus AMPKα2 were packaged in HEK293T cells (ATCC) by triple transfection. Large-scale plasmid preps of these packaging vectors were generated by Puresyn Inc. Briefly, 12 h before transfection, cells were seeded in 150 mm plates (30–50% confluent) fed with DMEM (Lonza) containing 10% fetal bovine serum (HyClone) with L-glutamine and penicillin/streptomycin (Gibco) (Li et al., 2019). After 48–72 h, cells were processed by TrypLE, collected in PBS (Corning), and resuspend in resuspension buffer (50 mM Tris-HCl pH 8.0, 150 mM NaCl, 2 mM MgCl2). The cells were subjected to three freeze-thaw cycles consisting of −80°C freezing for 10 min followed by thawing at 37°C for 20 min. Cells were incubated with 3,000 U Benzonase (Sigma) at 37°C for 1 h to digest cellular genomic DNA. The suspension was treated with 1/39th volume of 1M CaCl_2_ solution and 2/3 volume of 20% PEG 8000/1.25N NaCl to remove cell debris and precipitated adenovirus AMPKα2. Adenovirus were then resuspended in HBS and purified by CsCl_2_ gradient. After two rounds of CsCl_2_ gradient at 45,000 rpm and 60,000 rpm, fractions were collected according to the desired refractive index, respectively. Adenovirus were dialyzed against PBS in 10,000 MWCO Slide-A-Lyzer Cassettes (0.5–3.0 mL), and concentrated using an Amicon 100 kDa MWCO centrifugal filtration device (EMD Millipore Cat# UFC910008) prior to storage at −80°C(Chen et al., 2019a). Transfection of adenovirus into cardiomyocytes was conducted as previously described (Zhou et al., 2018a).

### Protein Extraction and Western Blotting

Protein lysates and western blotting were performed as described earlier (Hill et al., 2019). β-actin mouse monoclonal antibody (Cat. No. A5441, Sigma, St. Louis, MO) was used at 1:3000 dilution. Anti-rabbit secondary antibody from Millipore (Cat. No. AP307P, Darmstadt, Germany, 1:3000 dilution) and anti-mouse secondary antibody (Cat. No. 1706516, Bio-Rad, Hercules, CA) were used at 1:3000 dilution (Fardi et al., 2019). The protein bands were detected by chemiluminescence and quantified using Bio-Rad Image Lab 5.2.1 analysis software (Arun et al., 2018).

### RNA Isolation and qPCR

Total RNAs were isolated using an RNeasy kit (Qiagen, Hilden, Germany) following the manufacturer’s instructions (Bowman and Benet, 2019). Reverse transcription (RT) was performed using a QuantiTect Reverse Transcription kit (Qiagen) or a High Capacity cDNA RT Kit (Applied Biosystems, Foster City, CA). Both random hexamers and oligodT (Applied Biosystems) were used for RT reactions. Real-time quantitative PCR (RT-qPCR) was performed using SYBR green master mix (Applied Biosystems). GAPDH was used as an internal control unless specified otherwise (Bramasole et al., 2019).

### Measurement of Intracellular Reactive Oxygen Species

Dihydroethidium (DHE) and MitoSOX red mitochondrial superoxide indicator (Molecular Probes, United States) staining were performed as described (Zhang et al., 2019a), with some modifications. Before staining, cells were transfected with adenovirus-AMPKα2 for 48 h followed by addition of MitoSOX (10 umol/L) and incubated for another 30 min. Red fluorescence was visualized with a Zeiss microscope. In each experiment, images of five or six randomly selected fields were captured per sample (Chrifi et al., 2019). The resulting fluorescence was quantified using NIH ImageJ pro software and expressed as mean fluorescence intensity.

### Immunofluorescence Staining

Cells were fixed using paraffin. The sections were incubated with primary antibodies overnight at 4°C overnight and then with secondary antibodies for 30 min. For antibody specificity in immunofluorescence staining, isotype-matched normal IgG was used as the control for each assay. TUNEL staining was performed using a commercially available kit (Abcam ab83366) and (Roche 12156792910) (Huang et al., 2019). Images were acquired with a Leica immunofluorescence microscope.

### Intracellular Ca^2+^ Imaging

Characterization of intracellular Ca^2+^ dynamics was performed using Fura-2AM, as previously described (Jost and Hockendorf, 2019). Using a 488 nm laser for excitation (Curley et al., 2018), events were recorded at 20 to 50 fps with an EMCCD (Photometrics Cascade II 512, 512 × 512 pixels, 16-bit images) camera. Then, 10× (Olympus UPlanApo N.A. = 0.40) and 20× (Olympus UPlanSApo N.A. = 0.75) objectives were used for Ca^2+^ imaging (Frank and Vince, 2019).

### Statistical Analysis

All experiments were performed with three or more biological replicates unless mentioned otherwise in the figure legends. PRISM software (Graphpad, San Diego, CA) was used for data analysis. All data shown are the means ± SEM. *P* < 0.05 was regarded as statistically significant based on unpaired two-tailed *t*-tests (between two groups) or one-way and two-way ANOVA with Dunnett’s or Tukey’s multiple comparisons tests (between multiple groups). Normal distributions of data were confirmed using Shapiro–Wilk normality test. Kruskal–Wallis non-parametric test with Dunn’s multiple comparisons test and non-parametric Mann–Whitney test was performed for any data that did not pass the normality test.

## Results

### AMPK2α Overexpression Attenuates Hypoxia/Reoxygenation-Mediated Cardiomyocyte Dysfunction and Apoptosis

**FIGURE 1:**
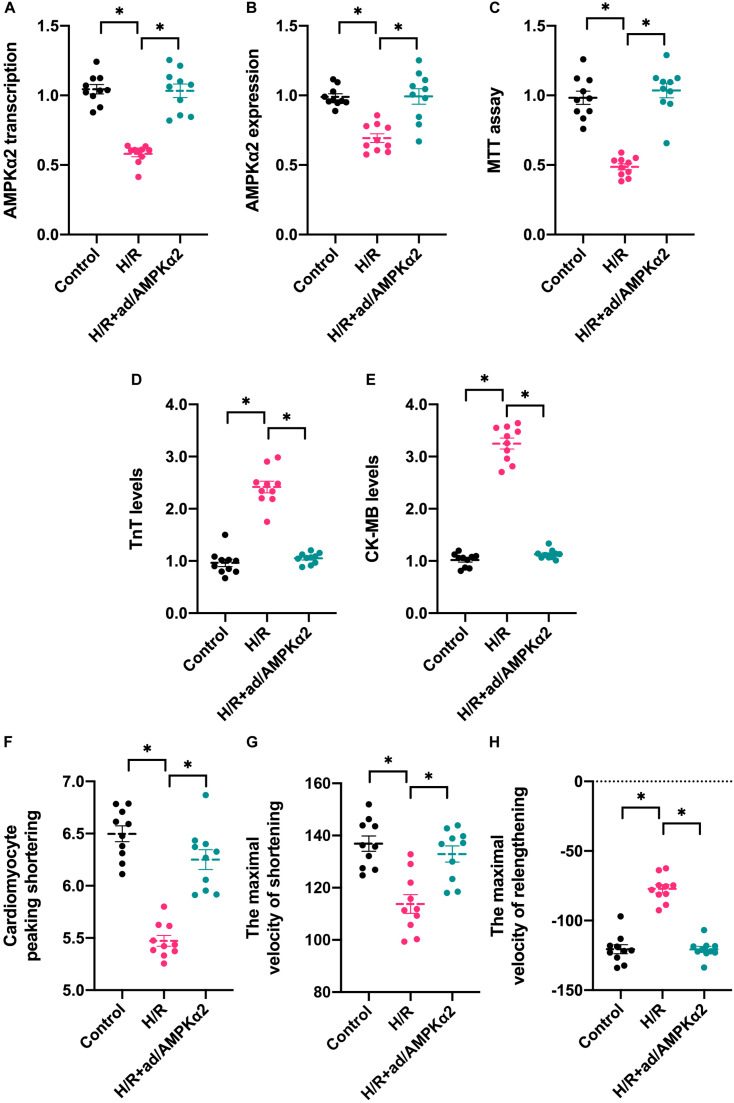
AMPK2α overexpression attenuates hypoxia/reoxygenation-mediated cardiomyocyte dysfunction and apoptosis. Adenovirus loaded with AMPKα2 were transfected into cardiomyocytes before H/R injury. **(A)** H/R injury was established through 1-h hypoxia, 2-h reoxygenation RNA was isolated from cardiomyocyte, and qPCR was used to evaluate the transcription of AMPKα2. **(B)** Proteins were collected from H/R-treated cardiomyocytes, and then, the expression of AMPKα2 was determined through western blots. **(C)** MTT assay was used to evaluate cell viability in response to H/R injury. **(D,E)** ELISA assay was used to measure the levels of troponin T (TnT) and creatine kinase-MB (CK-MB) in H/R-treated cardiomyocytes. **(F–H)** The cardiomyocytes contractile properties in the context of HI/R injury. The data represent the mean ± SEM. *P* < 0.05.

To understand the alterations of AMPK2α in response to hypoxia/reoxygenation (H/R) injury *in vitro*, RNA and protein were isolated from cardiomyocytes. Then, the transcription and expression of AMPK2α was determined through qPCR and western blots. As shown in Figure 1A, compared to the control group, H/R injury reduced the transcription of AMPK2α, and this finding was further supported through western blots (Figure 1B). To establish a link between decreased AMPK2α and cardiomyocyte damage under H/R injury, adenovirus-mediated AMPK2α overexpression was induced. The overexpression efficiency was confirmed through qPCR and western blots (Figures 1A,B). Then, cardiomyocyte viability was determined through MTT assay. As shown in Figures 1C, compared to the control group, cardiomyocyte viability was impaired by H/R injury whereas AMPK2α overexpression sustained cardiomyocyte viability. We also found that the levels of troponin T (TnT) and creatine kinase-MB (CK-MB) in the medium was upregulated after exposure to H/R injury, whereas this alteration could be reversed by AMPK2α overexpression (Figures 1D,E), suggesting that AMPK2α overexpression attenuates H/R injury-mediated cardiomyocyte damage. To observe whether AMPK2α overexpression could sustain cardiomyocyte function, we measured single cardiomyocyte contractions. As shown in Figures 1F–H, compared to the control group, cardiomyocyte peaking shortening, the maximal velocity of shortening, and the maximal velocity of relengthening were reduced after exposure to H/R injury. Interestingly, AMPK2α overexpression sustained cardiomyocyte contraction and diastole under H/R injury. Therefore, these data confirm that H/R-mediated cardiomyocyte damage is associated with a drop in AMPK2α levels.

### AMPK2α Overexpression Sustains Mitochondrial Homeostasis

**FIGURE 2:**
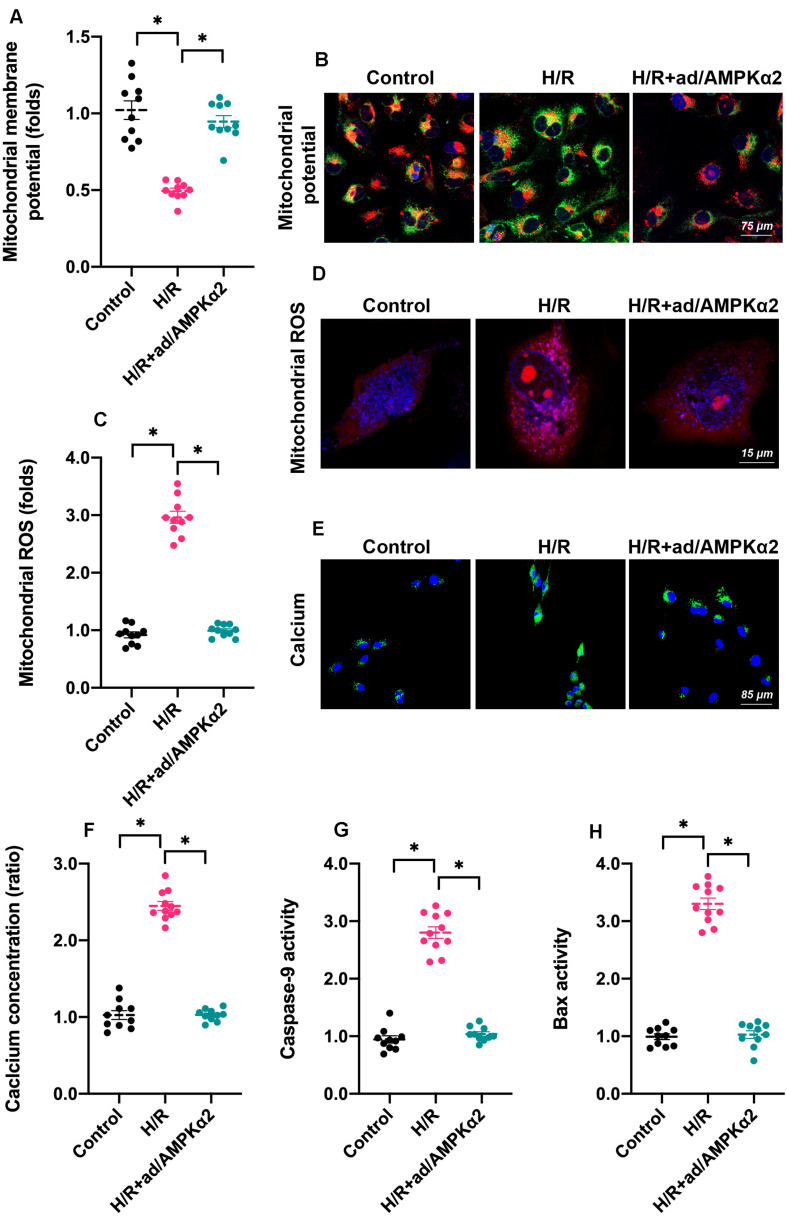
AMPK2α overexpression sustains mitochondrial homeostasis. Adenovirus loaded with AMPKα2 were transfected into cardiomyocytes before H/R injury. **(A,B)** Mitochondrial membrane potential was measured through JC-1 probe relative fluorescence intensity in H/R cardiomyocytes. **(C,D)** Intracellular ROS were determined through immunofluorescence. **(E,F)** H/R-mediated calcium overload was determined through immunofluorescence. **(G,H)** ELISA was used to measure the activities of caspase-9 and Bax in cardiomyocytes treated with H/R injury. The data represent the mean ± SEM. *P* < 0.05.

As we introduced above, mitochondria dysfunction has been identified as a major subcellular feature of cardiomyocyte damage during H/R injury. Given the beneficial effects afforded by AMPK2α overexpression on cardiomyocyte viability and function, we asked whether mitochondrial homeostasis could be sustained by AMPK2α. Firstly, we measured mitochondrial membrane potential since decreased electric potential energy is an early feature of mitochondrial damage (Berry and Wojtovich, 2020). Normal cardiomyocytes exhibited high membrane potential, which displayed bright red fluorescence (Figures 2A,B). After H/R injury, mitochondrial red fluorescence decreased whereas green fluorescence increased (Figures 2A,B), suggesting a drop in mitochondrial potential. Of note, AMPK2α overexpression drastically maintained mitochondrial potential (Figures 2A,B). At the molecular levels, mitochondrial potential reduction may be caused by increased mitochondrial membrane permeability whereas oxidative stress has been regarded as an independent risk factor for mitochondrial membrane hyper-permeability (Battistelli et al., 2019). Through ROS probe, we found that the levels of intracellular ROS were rapidly increased by H/R injury whereas AMPK2α overexpression prevented ROS overloading (Figures 2C,D). These effects may explain the protective effects exerted by AMPK2α on mitochondrial membrane potential. As a result of mitochondrial damage, intracellular calcium concentration was increased in response to H/R injury and this alteration could be inhibited by AMPK2α overexpression (Figures 2E,F). Lastly, we also noted that the activities of mitochondria apoptosis-related proteins, such as Bax and Caspase-9, were increased under H/R injury whereas AMPK2α overexpression prevented their activations (Figures 2G,H), suggesting that mitochondrial apoptosis may be blocked by AMPK2α overexpression.

### Mitochondrial Dynamics Are Disrupted by Hypoxia/Reoxygenation Due to Decreased AMPK2α Levels

**FIGURE 3:**
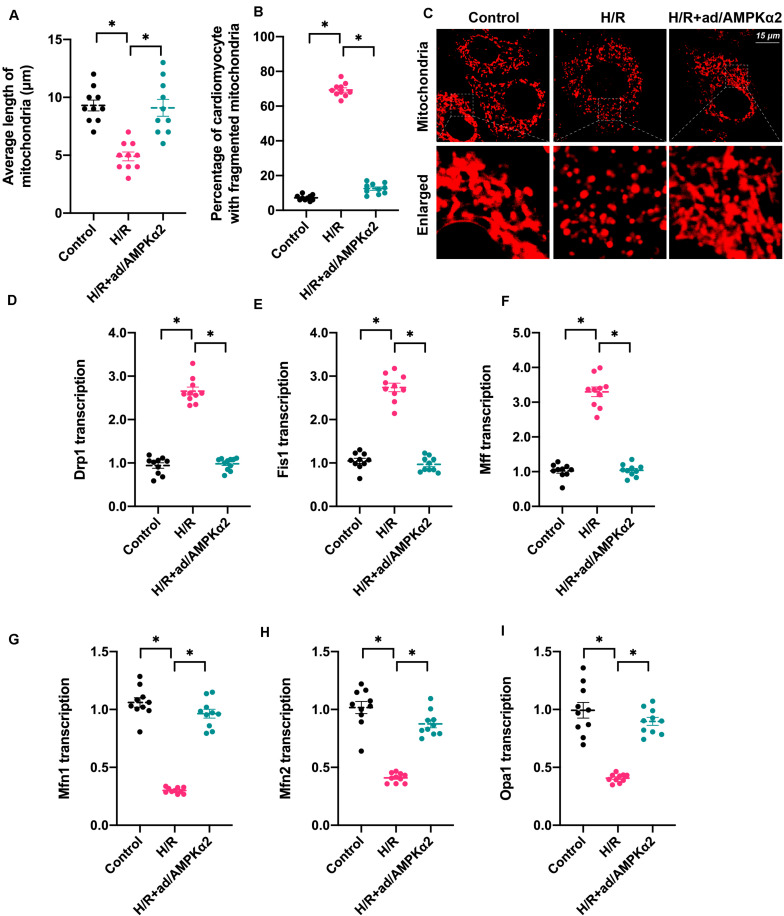
Mitochondrial dynamics are disrupted by hypoxia/reoxygenation due to decreased AMPK2α. **(A–C)** Mitochondrial morphology was measured through immunofluorescence. The average length of mitochondria as well as the ratio of cardiomyocyte with fragmented mitochondria was measured. **(D–I)** RNA was isolated from treated cardiomyocytes, and then the transcription of Drp1, Mff, Fis1, Mfn1, Mfn2, and Opa1 was measured. The data represent the mean ± SEM. *P* < 0.05.

Mitochondrial function is determined by mitochondrial morphology, which is regulated by mitochondrial dynamics (Li et al., 2020b; Wang et al., 2020b). Based on this, we asked whether mitochondrial dynamics could be regulated by AMPK2α in the setting of H/R injury. We firstly used immunofluorescence assay to observe changes in mitochondrial shape. As shown in Figures 3A–C, the predominant mitochondrial morphologies we observed were long strip whose average length was ∼9.8 μm. Upon H/R injury, mitochondria were fragmented and exhibited a shorter diameter with an average length of ∼4.8 μm (Figures 3A–C). AMPK2α overexpression sustained mitochondrial morphology and length in cardiomyocytes under H/R injury. This finding indicates that mitochondrial dynamics are disrupted due to decreased AMPK2α. Subsequently, RNA was isolated and genes related to mitochondrial dynamics were measured. As shown in Figures 3D–I, compared to the control group, the transcriptions of Fis1, Mff, and Drp1 were upregulated, whereas the levels of Mfn1, Mfn2, and Opa1 were downregulated, suggesting that mitochondrial division is activated whereas mitochondrial fusion is inhibited by H/R injury. Of note, AMPK2α overexpression was associated with normalized mitochondrial fission and improved mitochondrial fusion (Figures 3D–I). Overall, our results confirm that mitochondrial dynamics could be sustained by AMPK2α in H/R-treated cardiomyocytes.

### Induction of Mitochondrial Dynamics Disorder Abolishes AMPK2α Overexpression-Mediated Cardioprotection

**FIGURE 4:**
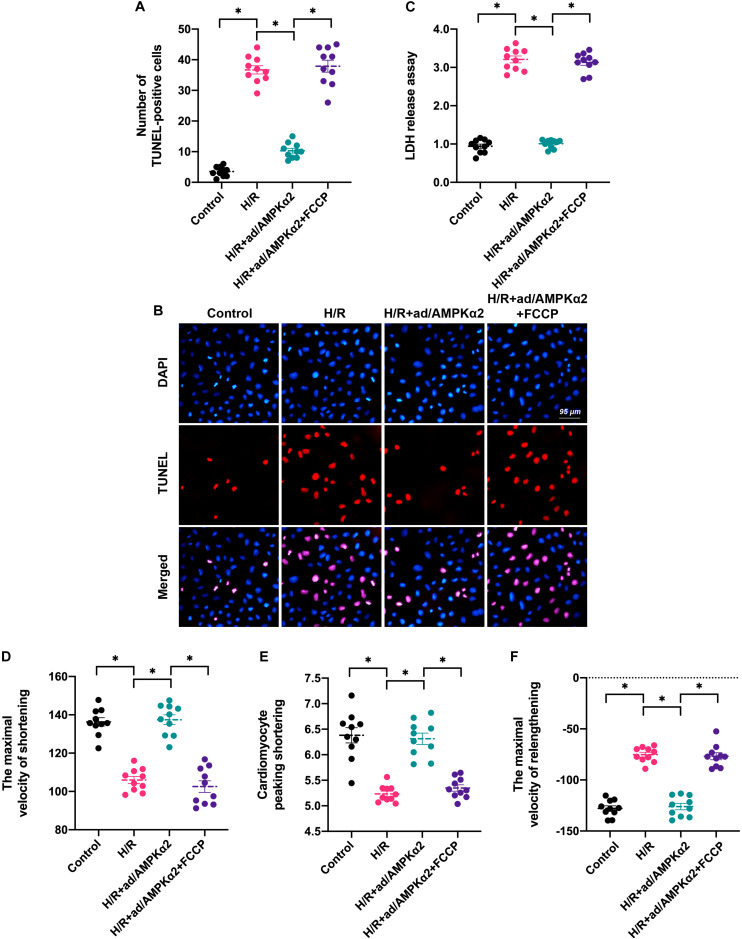
Induction of mitochondrial dynamics disorder abolishes AMPK2α overexpression-mediated cardioprotection. **(A,B)** TUNEL staining was applied to observe cell apoptosis. Cardiomyocytes transfected with AMPKα2 adenovirus were treated with FCCP to induce mitochondrial dynamics disorder. Then, the number of TUNEL-positive cells was recorded. **(C)** LDH release assay was used to measure cell viability. **(D–F)** The cardiomyocytes contractile properties in the context of H/R injury. The data represent the mean ± SEM. *P* < 0.05.

To test whether AMPK2α favors cardiomyocyte survival and contractility through normalization of mitochondrial dynamics, we incubated H/R injury cardiomyocytes with FCCP after transfection of AMPK2α-adenovirus. FCCP induces mitochondrial dynamics disorder through activation of mitochondrial fission and inhibition of mitochondrial fusion. We then measured cardiomyocyte viability and function using TUNEL staining and found that the number of apoptotic cardiomyocytes was augmented by H/R injury and this alteration could be attenuated by AMPK2α overexpression (Figures 4A,B). Of note, FCCP administration increased the apoptotic rate of AMPK2α-overexpressed cardiomyocytes (Figures 4A,B). In addition, LDH release assay also illustrated that AMPK2α overexpression prevented the LDH release caused by H/R injury whereas this effect was nullified by FCCP treatment (Figure 4C). Therefore, these data suggest that AMPK2α sustains cardiomyocyte viability through a mechanism involving normalization of mitochondrial dynamics.

With respect to cardiomyocyte function, cardiomyocyte contractility analysis demonstrated that cardiomyocyte peaking shortening, the maximal velocity of shortening, and the maximal velocity of relengthening were reduced after exposure to H/R injury (Figures 4D–F). Although AMPK2α overexpression sustained cardiomyocyte contraction and relaxation, its protective effects were thwarted by FCCP treatment (Figures 4D–F). Taken together, our results suggest that AMPK2α-mediated cardioprotection depends on normalized mitochondrial dynamics in the setting of cardiac I/R injury.

### AMPK2α Sustains Mitochondrial Dynamics Through the Sirt3/PGC1α Signaling Pathway

**FIGURE 5:**
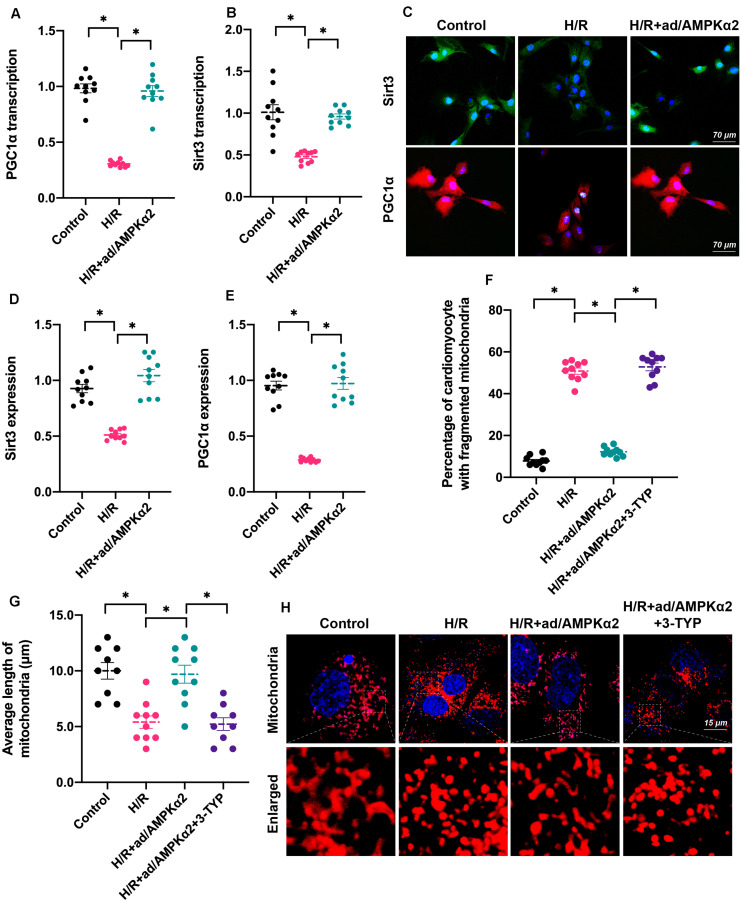
AMPK2α sustains mitochondrial dynamics through the Sirt3/PGC1α signaling pathway. **(A,B)** RNA was isolated from cardiomyocyte, and qPCR was used to evaluate the transcription of Sirt3 and PGC1α. **(C–E)** Immunofluorescence assay for Sirt3 and PGC1α in cardiomyocytes treated with H/R injury. **(F–H)** Mitochondrial morphology was determined through immunofluorescence. 3-TYP, an inhibitor of the Sirt3/PGC1α signaling pathway, was added into the cardiomyocyte medium before AMPK2α overexpression. The data represent the mean ± SEM. *P* < 0.05.

Lastly, we investigated the molecular mechanism underlying AMPK2α-controlled mitochondrial dynamics. Recently, Sirt3/PGC1α signaling has been identified as a key mediator of mitochondrial dynamics through upregulating or downregulating genes related to mitochondrial fission and fusion (Li et al., 2018; Sun et al., 2020). In addition, PGC1α also controls mitochondrial biogenesis and autophagy (Park et al., 2020; Wang et al., 2020a), contributing to mitochondrial turnover. Accordingly, we tested whether the Sirt3/PGC1α signaling pathway is under the control of AMPK2α. As shown in Figures 5A,B, RNA analysis demonstrated that Sirt3 and PGC1α were downregulated by H/R injury and reversed to near-normal levels with AMPK2α overexpression. Furthermore, protein analysis through immunofluorescence also illustrated that AMPK2α overexpression maintained intracellular Sirt3 and PGC1α levels in cardiomyocytes under H/R injury (Figures 5C–E). This finding supported the functional importance of AMPK2α on the stabilization of the Sirt3/PGC1α signaling pathway. To verify whether the Sirt3/PGC1α axis is required for AMPK2α-regulated mitochondrial dynamics, 3-TYP, an inhibitor of the Sirt3/PGC1α signaling pathway, was added into the cardiomyocyte medium before AMPK2α overexpression. Immunofluorescence assays for mitochondrial morphology showed that mitochondrial length was reduced by H/R injury compared to that of controls (Figures 5F–H). However, AMPK2α overexpression sustained mitochondrial length, but this effect disappeared after co-treatment with 3-TYP. Therefore, these results indicate that AMPK2α sustains mitochondrial dynamics through the Sirt3/PGC1α signaling pathway.

## Discussion

Ischemia-reperfusion (I/R) injury is a pathological process caused by the restoration of blood oxygen supply after ischemia and hypoxia, often accompanied by functional damage (Heusch, 2019). Earlier studies found that the main causes of cardiac I/R injury with incompletely defined mechanisms were inflammatory response, oxidative stress response, calcium overload, apoptosis, and no-reflow phenomenon mediated by the two phases of ischemia and reperfusion (Heusch, 2018; Meyer and Leuschner, 2018; Rossello and Yellon, 2018). At the same time, the release of reactive oxygen and nitrogen-containing substances after myocardial blood vessel transient occlusion can further induce tissue damage (Hadebe et al., 2018; Herzog et al., 2019). In the current study, we found that cardiac I/R injury seems to be associated with decreased AMPK2α. Decreased AMPK2α transcription was insufficient to sustain cardiomyocyte functions and was accompanied by disrupted mitochondrial dynamics. Abnormal mitochondrial morphologic alteration promoted mitochondrial dysfunction including mitochondrial membrane potential reduction, intracellular ROS/calcium overloading, and mitochondrial apoptosis activation. Further, our evidence supports a model in which AMPK2α sustains mitochondrial dynamics homeostasis through the Sirt3/PGC1α signaling pathway. This finding gives a novel insight into the pathogenesis of cardiac I/R injury and highlights the AMPK2α/Sirt3/PGC1α/mitochondrial dynamics signaling pathway as a potential therapeutic target for the treatment of cardiomyocyte damage caused by cardiac I/R injury.

Many studies have shown that mitochondrial dysfunction is central to cardiac damage during cardiac I/R injury (Zhou et al., 2017b; Zhu et al., 2018b; Wolint et al., 2019). For instance, mitochondrial unfolded protein response sends a cardioprotective signal for reperfused-heart and modulates cardiomyocyte protein homeostasis (Wang et al., 2019). Activation of mitochondrial biogenesis (Yang et al., 2019a) or promotion of damaged mitochondria removal (Zhou et al., 2020) also benefits damaged cardiomyocytes in the setting of cardiac I/R injury. Attenuation of mitochondrial stress by inhibition of mitochondrial fission favors cardiomyocyte survival under H/R injury *in vitro* (Ma and Dong, 2019). In light of the importance of mitochondrial integrity to cardiac health, several drugs and strategies have been developed to sustain mitochondrial homeostasis. Sodium thiosulfate attenuates mitochondrial ROS production and consequently reduces cardiac damage after cardiac I/R injury (Kannan et al., 2019). Protection of mitochondrial metabolism by hypothermia is useful to inhibit I/R-mediated cardiomyocyte dysfunction (Kohlhauer et al., 2019). Inhibition of cardiomyocyte sprouty1, a protein affecting mitochondrial morphology, is associated with a reduction of myocardial infarction zone and an increase in cardiac function after cardiac I/R injury (Alakoski et al., 2019). In the present study, we reported that mitochondrial dynamics disorder may be a novel feature of mitochondrial dysfunction. In accordance with our finding, a recent study also demonstrated that balancing mitochondrial dynamics through increasing mitochondrial fusion attenuates infarct size and left ventricular dysfunction in rats with cardiac I/R injury (Maneechote et al., 2019). Besides, improvement of mitochondrial fusion and mitophagy through administration of melatonin protects mitochondrial dynamics and cardiac function against I/R injury (Zhang et al., 2019b). Taken together, regulation of mitochondrial dynamics through promotion of mitochondrial fusion and inhibition of mitochondrial fission seem to be cardioprotective reperfusion strategies that warrant further clinical studies.

AMPKα2, the main subtype of AMPK, promotes mitochondrial metabolism, especially glucose consumption and fatty acid oxidation (Shires and Gustafsson, 2018), and seems to be linked to mitochondrial autophagy (mitophagy) (Wang et al., 2018). Damaged mitochondria are degraded by lysosomes to generate energy substrates such as amino acids and glucose, which are recycled by well-structured mitochondria to produce ATP with the help of AMPKα2 (Li et al., 2020a). Therefore, AMPKα2-mediated mitophagy is a metabolism-related molecular process. In the present study, we found that AMPKα2 promoted mitochondrial fission and inhibited mitochondrial fusion. This finding suggests a direct link between AMPKα2 and mitochondrial morphology, independently of metabolic alterations. These results provide new insights toward explaining the functions of AMPKα2 in mitochondrial protection. Previous studies have mainly reported the impact of AMPK, rather than AMPKα2, on mitochondrial morphology homeostasis. For example, hyperglycemia-mediated cardiomyocyte mitochondrial fission was attenuated by AMPK (Zhou et al., 2018d) while mitophagy was increased by AMPK in a cardiac I/R injury model (Zhang et al., 2019b). Further research is required to determine whether AMPKβ or AMPKα1 also regulate mitochondrial dynamics.

There are several limitations in the present study. First, due to technical problems, we didn’t use an AMPKα2 transgenic mice to verify the role of AMPKα2 overexpression in myocardial I/R injury *in vivo.* Second, mitochondrial dynamics also include mitochondrial biogenesis and mitophagy. Accordingly, further studies should explore the relationship between AMPKα2 and mitochondrial biogenesis/mitophagy in cardiac I/R injury.

## Data Availability Statement

All datasets presented in this study are included in the article/supplementary material.

## Ethics Statement

The animal study was reviewed and approved by Shunde Hospital, Southern Medical University (The First People’s Hospital of Shunde).

## Author Contributions

YD, SC, and YT designed and performed the experiments. JH, MZ, and CL collected the data and prepared the figures. All authors approved this submission.

## Conflict of Interest

The authors declare that the research was conducted in the absence of any commercial or financial relationships that could be construed as a potential conflict of interest.
